# Factors Associated with Meeting Physical Activity Guideline Recommendations in a Single Community Rehabilitation Center in Singapore: An Exploratory Study

**DOI:** 10.3390/healthcare14162648

**Published:** 2026-08-20

**Authors:** Llewelyn Yi Chang Tan, Lisa Wu, Chin Jung Wong, Jia Qian Goh, Matthew Rong Jie Tay

**Affiliations:** 1 Department of Rehabilitation Medicine, Tan Tock Seng Hospital, Singapore 308433, Singapore; 2 Department of Psychosocial Services, Singapore Cancer Society, Singapore 168583, Singapore; 3 Singapore Cancer Society Rehabilitation Center, Singapore Cancer Society, Singapore 168583, Singapore

**Keywords:** cancer rehabilitation, community rehabilitation, rehabilitation, exercise, physical activity

## Abstract

**Background:** Physical exercise is a vital component of cancer rehabilitation, with demonstrated improvements in cancer health-related outcomes, relapse rates, mortality and overall survival, yet global participation remains low. In Singapore, uptake of community cancer rehabilitation is limited despite high prevalence of treatment-related impairments. **Methods:** A cross-sectional study was conducted among adults (≥21 years) enrolled in the Singapore Cancer Society Rehabilitation Center between December 2021 and March 2023. Clinical data was collected from medical records and assessments included the Distress Thermometer (DT), Brief Illness Perception Questionnaire (Brief IPQ), and modified Bandura’s Exercise Self-Efficacy (ESE) scale. Adequate exercise was defined as ≥150 min/week of moderate intensity aerobic exercise and ≥2 days/week of resistance training. Regression analyses were performed to identify factors associated with meeting recommended moderate-intensity aerobic exercise levels. **Results:** The median age of our study cohort was 60.0 (IQR: 51.0–66.3) years. Of 132 analyzed participants, only 29.5% met recommended aerobic exercise levels and 9.1% met resistance training level recommendations. The median duration of moderate intensity aerobic exercise per week was 85.0 (30.0–180.0) minutes. The three most common cancer diagnoses amongst the participants were breast (53.8%), gastrointestinal (11.4%) and gynecological (7.6%) cancers. Clinically significant distress (DT ≥ 5) was present in 40.9% of patients. Multivariate regression analyses revealed that having lower ESE was the only significant psychosocial factor associated with a lower likelihood of meeting recommended aerobic exercise activity levels (OR = 0.856; 95% CI = 0.787–0.931; *p* < 0.001). **Conclusions:** Our study found a low prevalence of meeting recommended physical activity levels amongst our cohort of Asian cancer survivors enrolled in a community rehabilitation program in Singapore. Reinforcing the need for future longitudinal studies involving Asian cancer survivors to explore additional factors associated with physical inactivity and whether behavioral interventions targeting ESE will improve physical activity participation.

## 1. Background

Advancements in cancer diagnosis and management have led to improved survival rates through earlier detection and more effective treatments [1]. Despite these advances in cancer management, cancer treatment still results in significant short and long-term physical morbidity. This includes cancer-related fatigue, pain, neuropathies, cardiopulmonary limitations, balance and gait dysfunction [2,3,4,5]. Despite the high prevalence of these physical impairments, relatively few patients receive appropriate exercise-based rehabilitation, and referral rates to available cancer rehabilitation services have been reported to be as low as 1 to 2% [6].

Physical exercise is a vital component of cancer rehabilitation. Apart from improvements in physical function and aerobic fitness [7], physical exercise during cancer treatment has been associated with enhanced cancer treatment tolerability and a reduction in both the frequency and severity of cancer treatment-related side effects [8]. Physical exercise has also demonstrated improvements in cancer health-related outcomes including anxiety, depression, fatigue, physical function and health-related quality of life (QoL) [9,10,11,12,13]. Systematic reviews and meta-analysis have found that increased physical activity levels amongst cancer survivors are associated with reduced cancer relapse rates, reduced all-cause & cancer specific mortality, and improved overall [14,15,16]. As a result, numerous guidelines have recommended that all cancer patients avoid inactivity and engage in consistent physical exercise [17]. Most guidelines recommend that cancer patients aim for ≥150 min of moderate intensity aerobic exercise per week, along with resistance training ≥2 days per week for all major muscle groups, tailored to their specific diagnosis, treatment, and physical condition [17,18,19].

One key determinant of whether cancer survivors achieve adequate physical exercise is exercise self efficacy (ESE). Within Bandura’s Social Cognitive Theory (SCT) framework [20], self-efficacy is regarded as the most influential personal determinant of behavior change [21] and ESE refers to an individual’s confidence in their ability to exercise despite competing demands and barriers [22]. Unlike clinico-demographic characteristics, ESE is a modifiable construct that is amenable to targeted intervention.

Understanding how cancer survivors adopt and meet recommended physical exercise levels requires looking at both clinical associations and attitudes toward exercise. While clinico-demographic factors serve to identify who is likely to struggle to adopt physical exercise, attitudes reveal why they struggle and how interventions can be tailored to address these struggles. Key challenges in the local adoption of exercise-based rehabilitation services may include lack of awareness, passive engagement of community partners, poor health literacy, and worries about the risks of physical activity after a cancer diagnosis [23,24,25]. These can form barriers to patients being referred to or attending exercise programs in outpatient community cancer rehabilitation centers. Understanding these barriers is particularly crucial in community-based settings where patients have greater autonomy over their exercise choices compared to inpatient environments. Previous studies have predominantly focused on clinical predictors of exercise adherence in Western populations, but the underlying psychological mechanisms—particularly ESES and illness perceptions—remain understudied in Asian cancer survivors [26,27].

While Western studies have identified various predictors of exercise adherence, the applicability of these findings to Asian populations remains unclear. Community-based cancer rehabilitation programs have only recently been established in Singapore and other Asian countries, representing a shift from hospital-centric care models [13]. Cultural differences in illness perception, family dynamics, and healthcare expectations may influence exercise behavior differently in Asian cancer survivors. These psychosocial factors may be addressed through targeted interventions, making them critical targets for improving cancer rehabilitation program effectiveness.

Hence, it is important to identify cancer patients at risk of not meeting physical activity recommendations, as this will guide best practices to increase exercise adoption and help this vulnerable population reach recommended physical activity levels. As such, the primary objectives of this study were to identify the clinico-demographic and psychological factors independently associated with meeting physical activity guideline recommendations. The secondary objectives are to describe physical activity levels, characterize reported barriers to exercise, and examine attitudes toward exercise in this population. Establishing significant factors that are associated with meeting exercise requirements in Asian cancer survivors can inform future designs of community-based cancer rehabilitation programs.

## 2. Methods

### 2.1. Participants

This was a cross-sectional study that recruited cancer patients enrolled in the Singapore Cancer Society Rehabilitation Center between December 2021 to March 2023. This is a national community-based rehabilitation center, which receives referrals for cancer patients from clinical specialists or primary care physicians from any healthcare institution after they had started or completed their acute oncological treatment, e.g., surgery, chemotherapy, radiotherapy, hormonal therapy, immunotherapy, targeted therapy, or any combination treatment.

Referral criteria included cancer patients in the pre-treatment, active-treatment, or post-treatment phase who were medically stable and experienced symptoms requiring rehabilitative support. The community cancer rehabilitation program provides comprehensive exercise-based rehabilitation services with a physician-led multidisciplinary team, which includes physiotherapists, occupational therapists, nutritionists and medical social workers.

### 2.2. Inclusion and Exclusion Criteria

Eligibility included patients who are ≥21 years old and enrolled in the cancer rehabilitation program. Patients were excluded if they had terminally ill conditions, had major psychiatric illnesses or had cognitive or communication difficulties. This clinical study was performed in accordance with the principles of the Declaration of Helsinki and written informed consent was obtained from all the participants in this study. Ethical approval was obtained from the local institutional review board, Agency for Integrated Care (2021-007).

### 2.3. Assessment

After the first clinic visit with the cancer rehabilitation specialist, a one-time collection of data and assessments with questionnaires were performed during the enrollment of participants into the Singapore Cancer Society Rehabilitation Center’s cancer rehabilitation program.

The clinical and pathological data were collected through chart reviews, patient interviews and questionnaires. Data collected from patient electronic records included age, gender, ethnicity, comorbidities, type and stage of cancer and type of cancer treatment. Comorbidities were classified using the Charlson Comorbidity Index (CCI). CCI classes were defined as mild with scores of 1 to 2 points, moderate with scores of 3 to 4 points and severe with scores of ≥5 points [28,29].

The primary outcome measure studied was the achievement of the American Cancer Society’s (ACS) guideline of adequate aerobic exercise, defined as ≥150 min/week of moderate intensity aerobic exercise on average [17], based on the Physical Activity Vital Sign tool [30]. No patients achieved vigorous intensity of aerobic exercise. Aerobic exercise was defined as any form of sustained physical activity, encompassing aerobic activities such as brisk walks, jogging and participation in sports. We also studied the adequacy of resistance exercise per week, which was defined by the ACS as performing resistance exercise ≥2 days/week on average [17], specifically for descriptive and exploratory purposes. Resistance exercise was defined as any form of bodyweight exercises or resistance training performed involving major muscle groups [31].

The Distress Thermometer (DT) tool was utilized in our study. It is a single item self-reported measure of distress. Patients were asked to grade their distress in the past week on an 11-point visual analog scale ranging from 0 (no distress) to 10 (extreme distress). A DT score of ≥5 was used as a cut-off indicating a clinically significant level of distress [3,32].

The Brief Illness Perception Questionnaire (Brief IPQ) is a 9-item self-reported measure used to rapidly assess the cognitive and emotional representation of the participant’s illness based on a 11-point rating scale ranging from 0 (no illness perception) to 10 (greatest illness perception) [33]. These representations of illness include the consequences, timeline, personal control, treatment control, identity coherence, concern, emotional response and causes. Research has demonstrated the importance of illness representations to patient behavior [34]. In accordance with guidelines by the scale developers [33], the wording of the items was contextually adapted by substituting ‘illness’ with ‘side effects of cancer and treatment’. This adaptation was performed to specifically evaluate perceptions regarding treatment-related impairments, the primary target of cancer rehabilitation, rather than abstract disease state. Only the eight quantitative items (Items 1 to 8 of the Brief IPQ) were included in statistical summary and regression analyses.

Our study also utilized a modified version of Bandura’s ESE scale to assess ESE [20,35]. The modified ESE Scale by Neupert et al., itself is a modification of Bandura’s original ESE framework, for use with older adult populations [35]. The scale was administered in English, with no further modifications or deviations from the version described by Neupert et al. No formal cultural adaptation or prior pilot testing was undertaken for the current study population. The scale is a 9-item self-reported scale to measure how confident participants are regarding carrying out regular physical activities and exercise recommendations in diverse conditions and circumstances. Responses are based on a 4-point rating scale ranging from 1 (very likely) to 4 (very unlikely). A total score from the 9 items would be calculated and range from 9 to 36. A lower ESE total score of the 9 items would indicate higher ESE, while the highest score of 36 would represent the lowest ESE. The internal consistency (reliability) of the modified version of Bandura’s ESE scale was examined using Cronbach’s alpha (α) coefficient. A value of 0.70 or higher was deemed indicative of satisfactory internal consistency.

Common patient and societal barriers to exercise in cancer patients were also explored in our study [36,37]. Patients barriers explored include participants’ perception of whether they feel too weakened from their cancer therapy to participate in exercise, feel fatigued and have issues with sleep, being uninterested in an exercise rehabilitation program, being not physically active before their cancer diagnosis, having a fear of adverse events during the exercise rehabilitation program and not thinking that an exercise rehabilitation program will have a positive impact on their QoL. Societal barriers include high transport fees, daily stress, lack of time, and cost of such an exercise rehabilitation program [36,37]. Responses from participants were collected using a binary (Yes/No) format.

### 2.4. Statistical Analysis

Descriptive statistics of demographic and clinical variables were presented based on normality, as determined by the Shapiro Wilk test. Continuous variables with normal distribution were presented as mean and standard deviation, while those with non-normally distributed data were presented as median and 25 to 75th percentiles.

Logistic regression analysis was used to determine univariate associations (exposures) with meeting recommended levels of moderate intensity aerobic physical exercise (outcome). The following variables were subjected to univariate analysis investigating their relationship with meeting recommended requirements for aerobic exercise: age, gender, ethnicity, cancer type, cancer stage, treatment modalities (e.g., surgery, chemotherapy, radiotherapy, hormonal therapy, immunotherapy, targeted therapy), DT, illness beliefs, ESE, patient and societal barriers. In instances of complete or quasi-complete separation (e.g., zero cell counts in 2 × 2 cross-tabulations), Fisher’s exact test was applied to compute univariate values and Firth’s penalized likelihood logistic regression was applied to derive finite parameter estimates.

Candidate covariates for multivariable Firth’s penalized likelihood logistic regression were selected a priori based on clinical relevance and prior literature on physical activity factors in cancer survivors [38,39,40,41,42], rather than relying solely on univariable screening. The primary multivariable regression model was restricted to four core clinical and psychosocial regression coefficients: age (continuous), gender (male vs. female), cancer type (breast vs. non-breast), and ESE (continuous). Multivariate Firth’s penalized likelihood logistic regression was utilized given the relatively low events-per-variable (EPV) ratio. There were 39 outcome events (participants meeting aerobic exercise recommendations), corresponding to an EPV ratio of 9.75 (39 events across 4 estimated regression coefficients), which approaches the conventional minimum threshold of 10 EVP and increases the risk of small sample bias with standard multivariate logistic regression.

Statistical analyses were performed using IBM SPSS version 26.0 (IBM Corp., Armonk, NY, USA). All estimates were reported along with the 95% confidence interval (CI) and a *p* value of <0.05 was considered statistically significant for a 2-tailed test.

## 3. Results

A total of 133 cancer patients were recruited from the outpatient cancer rehabilitation clinic. However, 1 patient was excluded from data analysis due to incomplete data collection. (Table 1) The median age of our study cohort was 60.0 (IQR: 51.0–66.3) years. Most of the patients were 61 to 70 years old (31.1%) and 51 to 60 years old (27.3%). Females made up most of the recruited participants (78.0%), with most of the participants being Chinese (82.6%). The three most common cancer diagnoses amongst the participants were breast (53.8%), gastrointestinal (11.4%), and gynecological (7.6%) cancers. A significant minority of participants had metastatic disease (27.3%). Most patients had previous surgery (77.3%) and chemotherapy (75.8%), while a minority received radiotherapy (47.7%), hormone therapy (30.3%), and targeted therapy (3.0%). The median CCI score was 4.0 (IQR: 3.0–6.0) and most patients had severe (46.32%) comorbidity as measured with the CCI.

There were 54 (40.9%) patients who experienced clinically significant distress (DT scores ≥ 5). The scores of the Brief IPQ and perceived barriers to exercise are presented in Table 2 and Table 3. The most common personal barrier to an exercise-based rehabilitation program was problems with fatigue and sleep (27.3%). The most common social barrier was the impression that an exercise-based rehabilitation program was too expensive (31.1%). The median total score of the ESE scale was 22.5 (IQR: 18.8–26.0) (Table 4). Regarding exercise, the median duration of moderate intensity aerobic exercise per week was 85.0 (30.0–180.0) minutes. Only a minority of participants achieved at least 150 min of moderate intensity exercise per week (29.5%), with only 9.1% of participants achieving at least 2 days of resistance exercise per week.

Univariate logistic regression analyses revealed that patients were more likely to meet adequate aerobic exercise levels if they were older (OR = 1.041; 95% CI = 1.005–1.080; *p* = 0.025) had moderate comorbidity (OR = 4.299; 95% CI = 1.118–16.527; *p* = 0.034) and non-breast cancers (OR = 2.422; 95% CI = 1.124–5.150; *p* = 0.024). Significant factors such as being female (OR = 0.284; 95% CI = 0.120–0.670; *p* = 0.004), previous treatment with chemotherapy (OR = 0.429; 95% CI = 0.186–0.985; *p* = 0.046), lower ESE (OR = 0.840; 95% CI = 0.772–0.913; *p* < 0.001), the greater the experience of side effects of cancer treatment (OR = 0.842; 95% CI = 0.718–0.987; *p* = 0.034), the greater the emotional impact of the side effects of cancer treatment (OR = 0.821; 95% CI = 0.699–0.964; *p* = 0.016), the greater the perception that the side effects of cancer treatment have affected life (OR = 0.834; 95% CI = 0.717–0.970; *p* = 0.019) and the perception that the side effects of cancer treatment will continue (OR = 0.863; 95% CI = 0.748–0.996; *p* = 0.044) were associated with a lower likelihood of meeting adequate aerobic exercise levels. However, other factors such as age, ethnicity, DT score, presence of metastatic disease, previous surgery, previous radiotherapy, previous hormone therapy, previous targeted therapy, personal and societal barriers were not significant (Table 5).

A parsimonious multivariate Firth’s penalized likelihood logistic regression revealed that ESE was the only significant psychosocial factor associated with meeting physical exercise recommendations, where lower ESE was associated with a lower likelihood of meeting recommended aerobic exercise levels (OR = 0.856; 95% CI = 0.787–0.931; *p* < 0.001) (Table 6).

## 4. Discussion

This study characterizes the exercise levels, attitudes and perceptions among Asian cancer patients enrolled in a community-based rehabilitation program in Singapore. Increased exercise and physical activity can confer a multitude of health benefits to cancer patients [43]. These health benefits are known to also extend to patients with metastatic disease, making exercise interventions an integral part of every cancer survivorship program [13,44,45,46]. Despite the documented benefits of increased physical exercise in cancer patients, our study found a low prevalence (29.5%) of cancer patients fulfilling the recommended amount of aerobic physical activity per week with a smaller proportion (9.1%) meeting the recommended amount of resistance exercise per week. This is not dissimilar to other cancer studies, where it is estimated that only between 17% and 58% of cancer patients adhere to physical activity guidelines [47].

The low prevalence of cancer survivors meeting recommended aerobic physical activity levels in our community-based rehabilitation model reflects an international trend. Similar programs in Europe [48], Canada [39], Australia [49] and the USA [40] report comparable challenges with maintaining and meeting exercise targets, suggesting that the transition from hospital-based to community-based care presents universal implementation challenges. Sociodemographic, physical, physiological, and behavioral factors such as having more family support and feedback by trainers, high physical fitness, high self-efficacy, high motivation to exercise and being a non-smoker results in adherence to exercise interventions [38]. In Asian countries such as Japan [50], China [51], and South Korea [52], several studies have found other factors, such as family resilience, misconceptions about exercise resulting in cancer recurrence, poor psychosocial self-image and lack of clear knowledge, can result in poorer adoption and maintenance of exercise amongst cancer patients. Our multivariate analysis is consistent with the critical role of self-efficacy identified in Western studies, with ESE emerging as a significant independent factor associated with physical activity levels. Additionally, the barriers reported by our participants—fatigue, cost concerns, and time constraints—mirror both Western and Asian literature, indicating universal challenges that transcend cultural boundaries in community-based cancer rehabilitation.

Cancer patients with higher ESE tend to view exercise as a useful strategy in managing cancer-related side effects and demonstrate stronger intrinsic motivation and adherence to exercise. In contrast, patients with lower ESE show less initiative to use exercise to address cancer-related side effects, report poorer adherence to exercise, and experience lower levels of social support [26]. Our results are supported by previous findings where low ESE was associated with not meeting recommended levels of exercise [41]. Self-efficacy refers to a person’s confidence in successfully executing coping behaviors necessary to cope with cancer diagnosis and problems associated with it and its related treatment [20,53]. Self-efficacy varies depending on the context and the nature of the specific task and has become a target of many self-management interventions [54]. A higher self-efficacy in cancer patients has been shown to be associated with better wellbeing, QoL, and reduced physical and psychological burden [42,55,56,57]. ESE plays an important role in determining whether cancer patients initiate and adhere to exercise recommendations. Personal barriers such as cancer-related fatigue [26] and structural barriers, such as the lack of information or suitable exercise facilities [41] can influence ESE. Therefore, strategies to enhance self-management and overcome these barriers to exercise must be integrated into all cancer survivorship programs [58]. These self-management strategy programs center on empowering patients through education and fostering awareness among both patients and caregivers about the bidirectional relationship between physical activity and rehabilitation outcomes. Cancer survivors are taught how to take control of their own condition, with the goals of improving quality of life, boosting self-efficacy, and reducing reliance on health services.

Our multivariate analysis highlighted ESE as the only psychosocial factor associated with meeting physical exercise recommendations. However, this finding should also be interpreted with caution, as the relatively small sample size of our study may have limited statistical power, reducing our ability to detect independent associations for variables with smaller effect sizes after adjustment. Several variables were statistically significant in the univariable analyses but became non-significant after multivariable analyses. The loss of statistical significance after adjustment may be attributable to confounding, shared variance among analyzed factors, or model instability. Although several associations (e.g., gender, CCI, cancer type, previous treatment with chemotherapy, 4 components of the Brief IPQ) were not significant after adjustment, these associations can provide additional valuable exploratory insights to future studies and prompt clinicians to pay closer attention to these specific subgroups of cancer patients that may potentially be at risk of physical inactivity and warrant targeted interventions early.

Our study also revealed increased levels of illness perception within the cancer patients in the community, with median scores of the components of the Brief IPQ ranging from 5 to 7. A high proportion of our participants reported that the side effects of cancer and treatment had affected their lives and resulted in increased levels of distress. We postulated that these may have been attributed to the side effects and complications of chemotherapy, which was also found to be a negative association with achieving the recommended levels of exercise in our study on univariate analysis. The association between chemotherapy induced side effects and reduced level of physical activity is well reported in medical literature [46,59]. Chemotherapy treatment is known to result in a myriad of different side effects that may discourage cancer patients from exercising. These side effects include anemia, blisters, neuropathy, fatigue, gastrointestinal issues, myelosuppression, pain, and motor weakness [59]. Neuropathy related balance problems can lead to fear of falling and avoidance of exercise in public spaces without supervision. In a study by Toohey K et al., about one-third of participants reported that physical activity was not a priority when chemotherapy side effects predominated [60]. However, physical activity is beneficial in addressing side effects of chemotherapy including pain, fatigue, and neuropathy, which would otherwise lower physical activity levels [61,62,63]. This may reflect the relative lack of awareness amongst cancer patients regarding the benefits of exercise in addressing the side effects of chemotherapy and the potential lack of resources allocated in the community setting. This is evidenced by a local study in Singapore where only 46.1% of cancer patients reported receiving some form of exercise guidance from healthcare professionals following cancer diagnosis [62]. These findings provide supportive evidence for healthcare institutions to integrate early outreach and proactive screening programs as part of standard early oncological care. These interventions should also ideally be implemented at the point of cancer diagnosis as opposed to when patients are actively undergoing or have undergone cancer treatment and have reached states of physical inactivity.

Personal and societal barriers to exercise cited by our study participants included the lack of time (29.5%), cost issues (31.1%) and fatigue (27.3%). This is also supported by an earlier local study by Chan A et al. [64], where adverse effects from cancer treatment (52.0%), lack of time (20.6%) and costs (15.7%) have also been cited by cancer patients as barriers to physical activity [63]. Cancer treatment combined with the lack of adequate funding for community survivorship care can result in considerable financial strain on patients [23,65], and this may limit access to relevant exercise interventions [25,64]. Improving community-level cancer rehabilitation infrastructure requires a concerted effort and commitment from relevant stakeholders such as healthcare institutions, policy makers, and community organizations.

## 5. Limitations

There are several limitations that require highlighting. Firstly, the low-powered, single-center, exploratory, and cross-sectional nature of this study limits the generalizability and interpretation of results. Secondly, as study participants were already enrolled in our center’s rehabilitation program at the time of study recruitment, they may potentially be more motivated and engaged compared to cancer survivors not yet enrolled in such a program. Therefore, the reported associations in our study may not be generalizable to cancer survivors who are not yet enrolled in a structured community-based rehabilitation program. Thirdly, we were unable to obtain data pertaining to physical factors (e.g., baseline function prior to cancer diagnosis, frailty status at the time of study enrollment), socioeconomic factors (e.g., household income, mode of transport, presence of children, presence of a dedicated caregiver, social support), psychological factors (e.g., anxiety, depression) and treatment factors (e.g., details of chemotherapy regime), which may have also influenced physical activity and also confound our study’s observed associations, and we would like to highlight them as important variables for future studies to capture. Fourth, although ESE emerged as the only psychosocial factor retaining significance in our multivariable model, this finding should be interpreted cautiously. The modified version of Bandura’s Exercise Self-Efficacy scale used in this study was originally developed for older adults and, although its application to cancer survivors is plausible, its validity in this population has not been formally established. Furthermore, no formal cultural adaptation or psychometric validation was undertaken for our local Asian cancer survivor population. Therefore, our findings regarding ESE should be considered exploratory and require confirmation in future studies using validated instruments. Lastly, physical activity levels were all self-reported without objective verification and subject to recall bias, running the risk either under or overestimations.

## 6. Conclusions

Despite advances in cancer treatment and increased access to medical services over the years, our study found a low prevalence of meeting recommended physical activity levels amongst our cohort of Asian cancer survivors enrolled in a community rehabilitation program in Singapore. ESE was found to be the only factor independently associated with meeting recommended physical activity levels in the multivariate analysis. Future high powered longitudinal studies involving Asian cancer survivors are warranted to explore additional factors associated with physical inactivity and whether behavioral interventions targeting ESE, attitudes and barriers will improve physical activity participation.

## Figures and Tables

**Table 1:** Clinical characteristics of study participants.

Characteristics	*n* (%)/Median (IQR)
Age	60.0 (51.0–66.3)
Age 40 and below	11 (8.3)
Age 41 to 50	21 (15.9)
Age 51 to 60	36 (27.3)
Age 61 to 70	41 (31.1)
Age 71 and above	23 (17.4)
Gender	
Male	29 (22.0)
Female	103 (78.0)
Ethnicity	
Chinese	109 (82.6)
Malay	15 (11.4)
Indian	4 (3.0)
Others	4 (3.0)
Distress Thermometer (DT) Score	
<5	78 (59.1)
≥5	54 (40.9)
Charlson Comorbidity Index (CCI)	4.0 (3.0–6.0)
1 to 2 points (Mild comorbidity)	25 (18.9)
3 to 4 points (Moderate comorbidity)	46 (34.8)
≥5 points (Severe comorbidity)	61 (46.2)
Cancer Type	
Breast	71 (53.8)
Non-Breast	61 (46.2)
• Gastrointestinal tract	15 (11.4)
• Gynecological	10 (7.6)
• Lung	9 (6.8)
• Hematological	9 (6.8)
• Head and neck	7 (5.3)
• Genitourinary (Bladder/Prostate)	6 (4.5)
• Hepato-pancreato-biliary	2 (1.5)
• Other Ca or unknown primary	2 (1.5)
• Brain	1 (0.8)
Stage of Cancer	
Non-Metastatic	96 (72.7)
Metastatic	36 (27.3)
Treatment Received	
Surgery	102 (77.3)
Chemotherapy	100 (75.8)
Radiotherapy	63 (47.7)
Hormone therapy	40 (30.3)
Targeted therapy	4 (3.0)
Exercise Levels at Time of Recruitment	
Minutes of moderate intensity aerobic exercise per week	85.0 (30.0–180.0)
Adequate aerobic exercise ^a^	39 (29.5)
Adequate resistance exercise ^b^	12 (9.1)

Notes. IQR = Interquartile range. ^a^ Adequate aerobic exercise: ≥150 Minutes/week of moderate intensity aerobic exercise. ^b^ Adequate resistance exercise: ≥2 Days/week of resistance exercise of major muscle groups.

**Table 2:** Illness perception of study participants.

	Median (IQR)
How much does the side effects of cancer and treatment affect your life?	6.0 (4.0–8.0)
How long do you think the side effects of cancer and treatment will continue?	6.0 (4.0–8.0)
How much control do you feel you have over the side effects of cancer and treatment?	5.0 (3.0–7.0)
How much do you think a cancer rehabilitation program can help the side effects of cancer and treatment?	7.0 (5.0–8.0)
How much do you experience the side effects of cancer and treatment?	6.0 (4.0–8.0)
How concerned are you about the side effects of cancer and treatment?	7.0 (5.0–8.0)
How well do you feel you understand the side effects of cancer and treatment?	6.0 (5.0–8.0)
How much does the side effects of cancer and treatment affect you emotionally?	6.0 (5.0–8.0)

Notes. IQR = Interquartile range. Regarding scores from the Brief Illness Perception Questionnaire (Brief IPQ), higher scores indicate greater illness perception.

**Table 3:** Patient and societal barriers to exercise and rehabilitation.

	n (%)
Personal Barriers	
I feel weakened due to my cancer therapy.	16 (12.1)
I feel fatigued and have issues with sleep.	36 (27.3)
I am not interested in an exercise program.	6 (4.5)
I was not physically active before my cancer diagnosis.	21 (15.9)
I have a fear of adverse events.	8 (6.1)
I do not think an exercise program will have a positive impact on my quality of life.	5 (3.8)
Societal Barriers	
High costs of transport fees.	9 (6.8)
Stressful daily life.	15 (11.4)
Lack of time for exercise.	39 (29.5)
Rehabilitation is too expensive.	41 (31.1)

**Table 4:** Exercise self-efficacy of study participants.

	Median (IQR)
Exercise regularly (3 times a week, for 20 min).	2.0 (1.0–2.0)
Exercise when you are feeling tired.	3.0 (2.0–3.0)
Exercise when you are feeling under pressure to get things done.	3.0 (2.0–3.0)
Exercise when you are feeling down or depressed.	3.0 (2.0–3.0)
Exercise when you have too much work to do at home.	3.0 (2.0–3.0)
Exercise when there are other more interesting things to do.	2.0 (2.0–3.0)
Exercise when your family or friends do not provide any kind of support.	2.0 (2.0–3.0)
Exercise when you do not really feel like it.	3.0 (2.0–3.0)
Exercise when you are away from home (e.g., traveling, visiting, on vacation).	3.0 (2.0–3.0)
Total Score ^a^	22.5 (18.8–26.0)

Notes. IQR = Interquartile range. Scores from the modified version of Bandura’s Exercise Self-Efficacy (ESE) scale. (Cronbach’s α = 0.901). ^a^ Sum of scores from the 9 items of the modified version of Bandura’s Exercise Self-Efficacy (ESE) scale with the highest possible score of 36. The lower the total score, the higher the ESE the participant has.

**Table 5:** Univariate logistic regression for meeting adequate aerobic exercise levels.

Characteristics			
	OR	95% CI	*p* Value
Age	1.041	(1.005–1.080)	0.025
Gender			
Male	1.0		
Female	0.284	(0.120–0.670)	0.004
Ethnicity			
Chinese	1.0		
Malay	0.158	(0.019–1.247)	0.080
Indian	2.206	(0.298–16.322)	0.438
Others	2.206	(0.298–16.322)	0.438
Distress Thermometer (DT) Score			
<5	1.0		
≥5	0.634	(0.290–1.386)	0.253
Charlson Comorbidity Index (CCI)			
1 to 2 points (Mild comorbidity)	1.0		
3 to 4 points (Moderate comorbidity)	4.299	(1.118–16.527)	0.034
≥5 points (Severe comorbidity)	3.317	(0.884–12.448)	0.076
Cancer type			
Breast	1.0		
Non-Breast	2.422	(1.124–5.150)	0.024
Stage of Cancer			
Non-Metastatic	1.0		
Metastatic	0.889	(0.380–2.078)	0.785
Treatment Received			
Surgery	0.447	(0.192–1.040)	0.063
Chemotherapy	0.429	(0.186–0.985)	0.046
Radiotherapy	1.224	(0.579–2.588)	0.597
Hormone therapy	0.600	(0.254–1.419)	0.245
Targeted therapy	0.789	(0.079–7.833)	0.840
Illness Perception			
How much does the side effects of cancer and treatment affect your life?	0.834	(0.717–0.970)	0.019
How long do you think the side effects of cancer and treatment will continue?	0.863	(0.748–0.996)	0.044
How much control do you feel you have over the side effects of cancer and treatment?	0.922	(0.793–1.070)	0.293
How much do you think a cancer rehabilitation program can help the side effects of cancer and treatment?	1.116	(0.900–1.382)	0.316
How much do you experience the side effects of cancer and treatment?	0.842	(0.718–0.987)	0.034
How concerned are you about the side effects of cancer and treatment?	0.877	(0.763–1.010)	0.066
How well do you feel you understand the side effects of cancer and treatment?	0.988	(0.830–1.180)	0.894
How much does the side effects of cancer and treatment affect you emotionally?	0.821	(0.699–0.964)	0.016
Personal Barriers			
I feel weakened due to my cancer therapy.	0.513	(0.138–1.911)	0.320
I feel fatigued and have issues with sleep.	0.599	(0.245–1.466)	0.262
I am not interested in an exercise program.	0.180 ^b^	(0.012–1.841) ^b^	0.181 ^c^
I was not physically active before my cancer diagnosis.	0.708	(0.240–2.089)	0.531
I have a fear of adverse events.	0.323	(0.038–2.720)	0.299
I do not think an exercise program will have a positive impact on my quality of life.	0.221 ^b^	(0.013–2.417) ^b^	0.320 ^c^
Societal barriers			
High costs of transport fees.	0.664	(0.132–3.349)	0.620
Stressful daily life.	0.852	(0.254–2.859)	0.795
Lack of time for exercise.	0.761	(0.328–1.766)	0.525
Rehabilitation is too expensive.	0.690	(0.298–1.595)	0.385
Exercise Self-Efficacy (ESE)			
Total Score ^a^	0.840	(0.772–0.913)	<0.001

Notes. OR = Odds ratio; CI = Confidence interval. ^a^ Sum of scores from the 9 items of the modified version of Bandura’s Exercise Self-Efficacy (ESE) scale with the highest possible score of 36. The lower the total score, the higher the ESE the participant has. ^b^ Firth’s penalized likelihood logistic regression was applied. ^C^ Fisher’s exact test was used.

**Table 6:** Multivariate Firth’s Penalized Maximum Likelihood Logistic Regression for meeting adequate aerobic exercise levels.

Characteristics				
	β (SE)	OR	95% CI	Firth’s *p* Value
Age	0.021 (0.020)	1.021	(0.981–1.062)	0.300
Gender				
Male		1.0		
Female	−0.988 (0.576)	0.372	(0.121–1.150)	0.059
Cancer type				
Breast		1.0		
Non-Breast	0.196 (0.512)	1.216	(0.446–3.315)	0.699
Exercise Self-Efficacy (ESE)				
Total Score ^a^	−0.156 (0.043)	0.856	(0.787–0.931)	<0.001

Notes. OR = Odds ratio; CI = Confidence interval; SE = Standard error. ^a^ Sum of scores from the 9 items of the modified version of Bandura’s Exercise Self-Efficacy (ESE) scale with the highest possible score of 36. The lower the total score, the higher the ESE the participant has.

## Data Availability

The data presented in this study are available on request from the corresponding author due to privacy restrictions.

## Abbreviations

CCI	Charlson Comorbidity Index
DT	Distress Thermometer
Brief IPQ	Brief Illness Perception Questionnaire
ESE	Exercise Self-Efficacy
CI	Confidence interval
SCT	Social Cognitive Theory
QoL	Quality of Life
